# Areas of work-life that contribute to burnout among higher education health science faculty and perception of institutional support

**DOI:** 10.1080/17482631.2023.2235129

**Published:** 2023-07-18

**Authors:** Megan Koster, Kristen McHenry

**Affiliations:** Department of Respiratory Care, Boise State University, Boise, ID, United States of America

**Keywords:** Burnout, wellness, areas of work-life, support, higher education

## Abstract

**Background:**

COVID-19 added responsibilities to faculty in health-related fields. Educators in these areas have experienced pandemic-related role strain in both the clinical and academic settings.

**Purpose:**

This investigation sought to identify how health science faculty at one institution perceived challenges related to the COVID-19 pandemic in their role and to glean opportunities for institutions to increase the degree of support for faculty.

**Methods:**

An analysis of narrative comments was conducted on a survey assessing burnout and well-being. The survey was distributed to full-time faculty within the College of Health Sciences at a four-year institution. Using the areas of work-life model as a guide, two free-text questions within the survey were analysed to identify major themes.

**Results:**

39 participants contributed narrative responses to the qualitative, open-ended questions. Three themes emerged related to the areas of work-life categories: work-life imbalance, stress and unwellness, and unmet support needs. Strategies for enhanced well-being were noted to be workload management, administrative support, and wellness opportunities.

**Conclusions:**

This analysis provides insight into why health science faculty may be experiencing feelings of disengagement and exhaustion in their work. Enhanced workload and lack of community during the pandemic were major drivers of this phenomenon. Flexibility in workload, genuine concern and appreciation expressed by institutional leaders, and accessible wellness opportunities may help to offset these negative feelings.

## Introduction

Although the long-term effects of the COVID-19 pandemic on the workforce continue to be unravelled, there are likely several factors that have contributed significantly to the increased rates of workforce turnover (Cunningham et al., 2023; Lieder et al., 2023). These factors are presumably similar across fields, but there remain several areas wherein very little is known about the impacts of pandemic-related factors on job satisfaction. Higher education is still reeling from financial and pedagogical impacts, as well as institutional responses to the pandemic. As such, there is evidence that academic institutions are experiencing a higher-than-normal rate of faculty turnover (Flaherty, 2022). While there is speculation regarding specific causes for the exodus from academia, very little research has emerged regarding the perspectives of faculty teaching in the Health Sciences regarding the effects of teaching and learning through a global health pandemic.

Faculty at institutions of higher education are tasked with shaping the next generation of professionals within a specific field. In addition to these responsibilities, faculty members are also required to satisfy scholarly activity and service requirements. Although these expectations have been the status quo for academics for decades, it appears that the ability of an academic workload to sustain added pressures related to the COVID-19 pandemic caused even seasoned educators to experience symptoms of burnout. Burnout can be described as a lack of professional engagement and satisfaction (Awa et al., 2010). Although there has been plenty of emerging literature related to understanding burnout in general, very few studies have focused on the impact of burnout among healthcare faculty in higher education. This population is unique; in addition to classroom responsibilities, healthcare faculty are often responsible for facilitating the student experience in various domains including laboratory, simulation, and clinical environments. Additionally, it is not uncommon for educators within healthcare programming to perform dual roles; working not only in academia but continuing to practice clinically. Although the rationale for faculty who perform these dual roles may differ, reasons often range from maintaining a relationship with clinical affiliates, desire to remain grounded in practice, and/or financial need. This investigation aimed to identify how healthcare faculty at one mid-sized, four-year institution in the Mountain West region of the U.S. perceive challenges related to the COVID-19 pandemic on their role within higher education and to glean opportunities for institutions to increase the degree of support for faculty.

## Background

Over the last 15 years, stress levels in academic institutions have increased and remain higher when compared to other populations (Mark & Smith, 2012). The degree of burnout in this population may be related to the fact that the academic environment often requires faculty to fulfill competing roles which may have compounding responsibilities and add to levels of stress, both of which likely contribute to feelings of burnout. Abouserie (1996) found that 74% of higher education staff were moderately stressed and 15% were severely stressed. Minihan et al. (2022) reported that close to 82% of teachers reported experiencing burnout as a result of COVID-19. This is concerning outside of the contribution of burnout to turnover; sustained levels of increased or severe stress can lead to a multitude of issues both at the individual and systemic levels; many of which are health-related (Blix et al., 1994; Mark & Smith, 2012). There are several facets of work-life that contribute to feelings of burnout and diminished well-being; these include workload, control, reward, community, fairness, and values (Leiter & Maslach, 1999). Symptoms of burnout typically emerge when a mismatch occurs between the expectation, or needs, of individuals and the work environment across some or all these areas (Leiter & Maslach, 1999).

### Workload

Balancing work-life demands is difficult in any professional environment. However, this balance may be more problematic when a role requires the allocation of time to several different and distinct responsibilities. The personal coping strategies of faculty often include prioritizing work and striking a balance between teaching and research (Mark & Smith, 2012). Preparing in advance for their classes, seeking help when needed, and creating boundaries between roles were all methods used by faculty to support work-life balance (Mark & Smith, 2012). However, as impacts related to COVID-19 have underscored, drawing the line under the workday is difficult. Flaherty (2022) isolated several qualitative responses by university professors regarding their rationale for leaving the profession. Unsurprisingly, many respondents identified mismatches in work-life balance; indicating workloads that exceeded contract hours. Additionally, respondents indicated that stress, which impacted mental health and contributed to feeling unfulfilled, influenced their decision to leave the field (Flaherty, 2022). These themes were especially true for healthcare faculty; with a consensus that the stress of obligations throughout higher education was disproportionate to the severity of crises (Flaherty, 2022).

### Control

Lack of clear expectations within a role or objective can also lead to a sense of loss of control and can contribute to the rate of burnout (Barkhuizen et al., 2013). It is important for employees to have a degree of autonomy and to feel empowered by contributions to the institutional mission (Bennis & Nanus, 2007). Autonomy is typically linked to an increase in engagement, a desire for greater professional development, and less emotional depletion than those with less autonomy (Barkhuizen et al., 2013; Bennis & Nanus, 2007). For faculty who were tasked with facilitating clinical encounters for students, the experiences of working through the COVID-19 pandemic may have been even more unique. Responsibilities specific to these faculty include detailed coordination with clinical affiliates, managing multiple clinical policies and requirements, communicating with students to submit documentation, and scheduling learning opportunities. Under normal circumstances, these responsibilities are time-consuming, and successes are highly contingent upon the clinical environments; meaning that a faculty member may not always have complete control over clinical coordination.

When organizational change happens quickly, as was the case throughout the COVID-19 pandemic, occupational stressors increase the stress response in employees (Day et al., 2017; Gerding et al., 2021). Gerding et al. (2021) found that 80% of healthcare workers experienced an increase in workload as a result of the COVID-19 pandemic; a marked increase compared to other professions. This added workload contributed to the degree of burnout experienced by workers and was associated with higher rates of sleep deprivation as well as disengagement (Tamrakar et al., 2021). These effects are likely compounded by the loss of control or autonomy-related diminished clarity surrounding objectives, desired outcomes, or simply an unstable environment throughout the change. Understanding the dynamic between engagement and locus of professional control is important because lack of control has been associated with disengagement, which is a precursor to burnout (Barkhuizen et al., 2013). Although many healthcare workers, academics, or the hybrid academic-healthcare faculty are often described as flexible individuals used to navigating a dynamic environment, external global or societal factors, like those related to COVID-19 May 2001have exacerbated issues relate to control due to the increased time required to navigate unclear roles and/or responsibilities (Cunningham et al., 2023).

### Reward

The term “reward” is multifaceted in the sense that there are several types of rewards for work performed. Individuals may seek intrinsic and extrinsic rewards, or both, related to professional accomplishment. Rewards are also contingent upon institutional culture and are heavily influenced by perception and equity among rewards (Leiter & Maslach, 1999). These perceptions often influence the extent of effort put towards an objective. It is important that there be a balance between the perceived effort put forth and the type of reward received; if there is a mismatch, either real or perceived, it may lead to disengagement, which again contributes to symptoms of burnout (Leiter & Maslach, 1999).

Disengagement as a result of unmet needs, or perceived inequitable rewards, is challenging to differentiate among individuals. However, faculty with fewer rewards and less recognition who also experience an increase in work demands, energy, and time run a greater risk of becoming more exhausted and alienated from their work (Barkhuizen et al., 2013). Although there has been a positive correlation between intrinsic reward and job satisfaction, it is imperative that institutions understand that there is likely a tipping point for the allocation of effort and system of reward (Mark & Smith, 2012). Additionally, there exists a phenomenon of “overcommitment” throughout both the healthcare and educational fields. Sérole et al. (2021) identified feelings of responsibility and duty related closely to overcommitment, making it difficult for healthcare professionals to set healthy boundaries. Subsequently, there appeared to be a relationship between feelings of inadequate reward for time committed to work.

### Support

Another key factor related to job satisfaction is the degree of institutional and social support (Barkhuizen et al., 2013). In a 2022 survey of College and University Chief Academic Officers, approximately 60% of Provosts expressed concerns about the degree of support faculty perceive from the administration. However, 51% of Provosts who responded indicated that there was ambivalence towards taking concrete steps to address faculty burnout (Jaschik, 2022). Institutional support can be integral to buffering employees from stressors related to change which typically contribute to cynicism and/or exhaustion (Day et al., 2017). Even prior to the COVID-19 pandemic, Barkhuizen et al. (2013) suggested employers focus on management relations, role clarity, and autonomy as ways to demonstrate support. Employees who have supervisors who are supportive, consistent, and show appreciation are often more engaged, which can lead to a decrease in burnout (Barkhuizen et al., 2013). This is likely due to the reciprocation in value for effort; meaning, individuals who feel valued and supported by an institution typically contribute more effort towards a desired outcome than do individuals who do not feel empowered by their employer (Barkhuizen et al., 2013; Bennis & Nanus, 2007). The remaining three areas of work-life (community, fairness, and values) can all be represented through the presence, or lack thereof, of institution-specific support.

### Wellness

Wellness is often described as being in a state of good health. For the purposes of this investigation, wellness is operationalized to encompass mental, physical, and emotional health. Each component of wellness is associated with an individual’s perception of happiness and satisfaction at a different level. However, as levels of either disengagement or exhaustion rise, the perception of overarching wellness decreases. Wherein most people undergo infrequent traumatic experiences with time to recover, healthcare professionals typically do not have time to recover from one traumatic occurrence before addressing another (Bays, 2022). This “chronic acute stress” contributes to a degradation of emotional reserve and leads to both exhaustion and disengagement (Bays, 2022, p. 4). Navigating professional expectations and the needs of students is a heavy burden. Healthcare educators, in addition to carrying the emotional and physical stress of their own practice, often carry the emotional burden of their students’ experiences. This phenomenon has been difficult to quantify outside of an individual organization; however, additional stress related to these roles may compound or exacerbate existing stressors and contribute to a perception of overall wellness.

## Methods

This paper examines and reports only the qualitative findings of a survey that assessed burnout and well-being in higher education health science faculty. The quantitative portion involved collecting data that incorporated the Oldenburg Burnout Inventory (OLBI) and World Health Organization-5 Well-Being Index (McHenry et al., 2023). Subsequently, after assessing the findings related to burnout and well-being, qualitative responses to two questions were reviewed to better understand the reasoning for the perceived burnout, similar to a study conducted by Miyasaki et al. (2017). These questions asked respondents to reflect on and describe areas of work-life that contributed to burnout and wellness strategies that respondents have employed during the pandemic or would like to begin implementing with support from their institution. Though the responses from both quantitative and qualitative questions were obtained concurrently, the data were analysed consecutively. The rationale for this study design was to extrapolate why faculty members may be experiencing burnout and report tangible ways these feelings could be prevented or mitigated through a shared responsibility of the individual and the institution.

The study protocol was granted Institutional Review Board (IRB) approval on 5 May 2021. The survey was open from June 4 through 30 July 2021. One hundred and seventeen full-time faculty members in the College of Health Sciences at a mid-sized four-year institution were surveyed using a web-based tool, and participation was incentivized with a gift card. Frequent reminders were also sent to those who had not completed the survey. Faculty were excluded if they did not serve full-time (18–30 credit hours per academic year depending on position/rank), were classified as adjunct faculty, or if they participated in the pilot survey. Faculty stemmed from various programmes including nursing, community and environmental health, kinesiology, social work, respiratory care, genetic counselling, and radiologic sciences. Of the one hundred and seventeen full-time faculty who met the inclusion criteria, forty-five (38.4%) completed the survey. Free text analysis of two open-ended questions was then coded into areas of work-life and analysed to identify major themes regarding the perceptions of well-being and institutional support which may have contributed to burnout in this population.

### Data Analysis

Initial data were collected via a web-based survey tool. Open-ended questions were included to garner additional information related to factors that contributed to the rate of burnout and diminished wellness. The qualitative responses central to this investigation were hand-coded, categorized into expected predetermined themes (i.e., areas of work-life), and assessed for additional emerging areas of interest. This combination of using predetermined and emerging themes is common in qualitative health science research when a model or theory currently exists (Creswell & Creswell, 2018). Reliability of the analysis was sought through coordination among the researchers, which included cross-checking during the coding process and ensuring intercoder agreement. Member checking also took place through the reporting of findings during presentations at open forums specifically in the schools of allied health and nursing to aid in the validity of findings.

## Results

**Figure 1.**
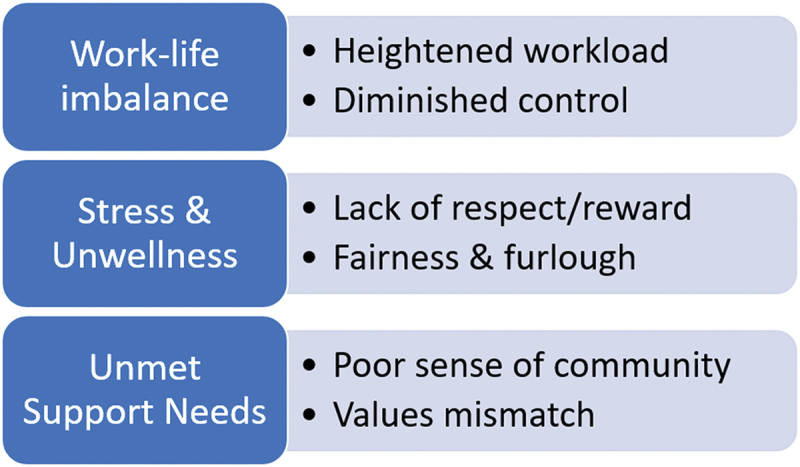
Relationship between areas of work-life and emerging themes.

Of the one hundred and seventeen full-time faculty who met the inclusion criteria, forty-five (38.4%) completed the survey, and at least thirty-nine (33.3%) contributed narrative responses to the qualitative, open-ended questions. Results of the quantitative data indicated a moderate to high level of burnout experienced by this sample of faculty during the pandemic which was associated with a poorer self-reported sense of well-being. Faculty with clinical teaching responsibilities reported higher rates of burnout. Complete findings of the quantitative portion of the study are reported elsewhere (McHenry et al., 2023). The predetermined themes were interwoven throughout the narrative responses. Figure 1 provides the visual connection between the areas of work-life categories and the emerging themes.

A heightened workload in conjunction with an apparent lack of control and reward during the pandemic may have contributed to a real or perceived work-life imbalance. Feelings of work not being valued and healthcare disciplines not being respected may have impacted stress levels influencing the respondents’ sense of wellness and well-being. A fractured sense of community, both institutional and social, coupled with a potential values mismatch, led respondents to feel undersupported during a time in which they could have benefited most. The majority of responses stemmed from Allied Health Sciences (*n* = 17), Nursing (*n* = 14), and Community and Environmental Health (*n* = 6), with Social Work and Other programmes having one response each (*n* = 2). Faculty who contributed qualitative responses were considered clinical track (*n* = 13), tenured (*n* = 12), tenure track (*n* = 9), or instructor (*n* = 4). One respondent left this question unanswered. Most faculty reported 9-month contract appointments (*n* = 21) or 10 to 12-month contracts (*n* = 18). Table I outlines the thematic classification of qualitative responses to the inquiry regarding specific work-life areas perceived as more challenging in 2020–2021.

**Table I.** Thematic constructs: Have you found any areas of work life more challenging in the 2020–2021 school year? Please explain.

Theme	Response
Work-Life Imbalance	*“Balancing work and parenting demands. I’ve had kids at home all year and it’s hard to strike a balance some days”* *“Balancing kids in online school and meeting requests”* *“Everything was a bit more challenging with the pandemic and all the changes it precipitated. Students were in need of greater support and were more stressed, colleagues were more stressed, everyone was unsettled and a bit fearful”* *“ … Balancing work with constant interruptions of family made it really hard to maintain concentration, quality work, etc.”* *“Boundaries - when to stop working while at home”*
Unmet Support Needs	*“It has been more challenging to stay engaged with my teammates. With working at home, the hallway conversations are gone, and I forget to bring up some topics when I do get to talk with my teammates”* *“Dealing with the administrators…It seems like their intent is to make our jobs harder instead of finding ways to help us do our work”* *Lack of contact with colleagues made communication and problem-solving more difficult*. *Using ZOOM technology, while efficient, also lacks the ‘connection’ that personal meetings have. This seems to have contributed to more feelings of being isolated from coworkers as well as more frequent communication problems (usually b/c of the shift in complete reliance on email/zoom versus ‘in person’ communication*
Unwellness	*“ … I am so tired. I just don’t know if that I could continue “last year” for very long without giving up”* *“Mental wellness”* *“Work was no longer enjoyable, it caused real, physical, and emotional symptoms”* *“Furlough days despite having an increase in the amount of time and mental energy required due to COVID issues”* *“It’s challenging to feel like your work is valued when budget cuts force faculty to incur furlough days and the subsequent decrease in pay for those days”* *“Colleagues were more stressed, everyone was unsettled and a bit fearful”*

A second question asked respondents what types of wellness strategies the institution could provide to support faculty during this time better. Three main themes emerged and corresponded to the analysis of the preceding question. Respondents indicated that wellness strategies offered by the institution should address workload, support, and wellness. Table II outlines the thematic classification of responses related to this question.

**Table II.** Thematic constructs: What wellness strategies could the institution provide to support faculty? Describe.

Theme	Response
Workload management	*“A more reasonable workload, so I don’t feel like I have to work a couple weekends a month to stay afloat* *“Better role modeling by leaders; taking time off that is due a person; not saying “take time off” but piling plates so full that people can’t take time off; true time off with NO communication”* *“Permanent flex/work from home ability; Zoom-free/meeting-free days; Reduce committee work or non-essential initiatives like strategic planning during a pandemic when faculty are mentally exhausted”* *“Potentially offering a class on time management or organizational tips would be great. There never seems to be enough time in the day”*
Administrative Support	*“Providing a confidential space to connect with other faculty experiencing the same stresses … Being able to hear that other faculty are experiencing the same thing helps you feel less alone. It also opens the door to sharing how they are dealing or not dealing with it”* “*Appreciation events, showing up to class and thanking faculty”* *“Higher wages”* *“Childcare, therapy”* *“Honest and inclusive communication”*
Wellness Opportunities	*“Provide more approved wellness hours during work hours. Currently, we have one per week; schedule activities throughout campus instead of one setting which may be too far for some faculty and staff”* *“Free access to the recreation center”* *“Have our health insurance cover massages. Massages have a lot of health benefits”* *“Family-friendly wellness events (e.g., fitness, cooking, yoga, mindfulness) with provided childcare”* *“Support group facilitation-nutrition or exercise or meditation, etc.”* *“I think that faculty should be provided with memberships to the rec center as part of their reimbursement package. I suspect that there are more wellness strategies offered than I am aware of; more intensive communication of strategies available for faculty would be good”*

## Discussion

The survey results from the qualitative questions were consistent with other studies conducted with faculty at higher institutions. In regard to workload, respondents indicated that what was expected to be performed during this tumultuous time was a major contributor to the perceived feelings of either disengagement or exhaustion. Workload allocation shifted disproportionately towards focusing on students’ needs and their wellness, which was difficult to anticipate and tangibly measure. Respondents also felt that the time and energy related to supporting students contributed to less time and/or energy to perform other job-related tasks and added an emotional burden to their professional responsibilities.

At the time of this investigation, at the height of the initial COVID-19 pandemic, institutional administration provided very little relief in the form of workload redistribution. Meaning that individuals were still required to fulfill contractual obligations related to teaching, service, and in some cases, scholarly activity with little consideration for the additional responsibilities incurred. Because faculty were already operating at 100% of workload capacity at the time of the pandemic’s onset, there was little these faculty could do to buffer impacts on workload related to the COVID-19 pandemic. Respondents indicated that the institution should seriously consider more flexible workloads, opportunities to work remotely, and allow faculty to triage non-essential tasks to focus on critical workload items. Empowering faculty to provide feedback on the allocation of workload, or redistribute their energy according to essential priorities may help to bolster the autonomy and control which are central to job satisfaction (Boamah et al., 2022).

Mark and Smith (2012) identified a positive correlation between high levels of anxiety with increased job demands and loss of control. High levels of depression were also associated with high job demands, intrinsic and extrinsic efforts (Mark & Smith, 2012). There were factors relating to generalized exhaustion within the existing workload of these specific individuals which were exacerbated by events related to COVID-19. Several respondents indicated that they had considered leaving the institution. This reaction is consistent with other literature on the topic. Boamah et al. (2022) indicated that turnover intent, described as the perceived likelihood of impending departure from a job or institution, is the last stage of disengagement. This is an important variable for institutions to monitor, as turnover related to disengagement and/or burnout compounds an existing perception of inadequate workload expectations and may result in a higher degree of attrition (Boamah et al., 2022).

In a 2022 survey of College and University Chief Academic Officers, approximately 60% of Provosts expressed concerns about the amount of support faculty perceive from the administration (Jaschik, 2022). However, 51% of Provosts who responded indicated that there was ambivalence towards taking concrete steps to address faculty burnout (Jaschik, 2022). This degree of ambivalence may explain the perception of unmet support needs as a primary theme identified in this sample. Respondents indicated frustration with the administration which centred on the mismatch of expectations. For example, faculty served as the primary point of contact for students in crisis or in need of additional help, but there was no reallocation of workload available to help faculty reconcile their student-focused efforts with other job requirements. Additionally, although the institution worked to provide opportunities for faculty and staff to engage in wellness activities, many of those opportunities were during dates and/or times which severely limited access/availability. Several respondents indicated that rather than creating one-off opportunities, the institution should invest in sustainable and proactive support systems for faculty on a regular basis. For example, faculty respondents requested a discounted or free membership to the University’s recreation centre. Other suggestions included tangible resources such as allocations towards on-site childcare or therapy options.

Several respondents lamented the loss of social and/or professional support systems during the COVID-19 pandemic. Literature has emerged related to the levels of stress and anxiety perceived by faculty in higher education because of impacts on their professional structure. VanLeeuwen et al. (2021) highlighted that many faculty were experiencing high levels of stress and anxiety due to the extra hours required of them to perform job tasks; many of which were related to students, rather than contractional obligations, like research. Contributing to this was the emergent shift from traditional face-to-face teaching to an online learning environment. Although institutional support was made available for faculty who were not familiar with this modality, the learning curve was steep and likely contributed to additional burdens (VanLeeuwen et al., 2021). This, paired with the perceived loss of collegial support by way of isolation, likely contributed to how faculty perceived the level of support offered by the institution.

Additionally, the disruption of both personal and professional routines may have resulted in feelings of both sadness and loss among faculty. The emergent shift towards a virtual environment for not only teaching, but interaction in general, likely contributed to feelings of isolation and loss among faculty. Respondents specifically requested opportunities for group counselling, stating that the need for a confidential space to connect with other faculty to create a sense of shared experiences would have helped them address the feelings of isolation which may have contributed to feelings of disengagement. Mark and Smith (2012) identified that improving social support systems, enhancing rewards systems, training in problem-focused coping methods, and how recognizing the dangers of becoming overcommitted to work improved overall performance. Support networks are essential to maintaining the effort/reward balance; organizations that incorporate strategies to assist their employees in managing and coping with daily responsibilities may have a higher degree of retention and employee satisfaction.

The overwhelming shift in responsibilities of these faculty to not only ensure that students could navigate the challenges relating to COVID-19 while meeting academic requirements but also navigate their own challenges with limited social connectivity seems to have had an impact on the perception of overall wellness. Although the quantitative results of this investigation identified that this sample perceived themselves as generally “well” (McHenry et al., 2023); there were several qualitative responses that indicated a subsample of faculty who were struggling. An assessment of the degree to which the greater population of higher education faculty has been affected by impacts related to the COVID-19 pandemic has been slow to emerge. However, the initial qualification of faculty wellness is concerning. Melnyk et al. (2021) highlighted in a study of roughly 2,200 faculty that 18–27% of faculty met the cut-off for clinical anxiety and as many as 8.3% of faculty were depressed.

Coping mechanisms may vary across individuals, Melnyk et al. (2021) quantified the top three most common mechanisms for faculty coping including connecting with others, exercise, or being in the service of others. However, Melnyk et al. (2021) also reported an increase in unhealthy behaviours because of confinement and loss of routine, including an increase in alcohol consumption and consumption of unhealthy food. The responses in this investigation regarding opportunities for increased institutional support centred on wellness were consistent with existing literature; specifically, the access to mental health resources and the need for proactive and sustainable resources related to physical fitness (Melnyk et al., 2021).

The clear perception that work was not valued indicated that some respondents had met the point at which the dedication of effort was no longer congruent with the rewards experienced, regardless of the intrinsic motivation. The institutional use of mandatory furlough days based on salary was likely a contributing factor to faculty exhaustion and disengagement and resentment towards workload and likely underscored the respondents’ perceived feelings that there existed a lack of institutional support. This surfacing desire for benefits that support tangible aspects of workload, support, and wellness, rather than financial incentives, is an interesting phenomenon and one which underscores the potential investments institutions can make to better support faculty in the future. It is imperative that institutions begin to view employee wellness as an investment to combat symptoms of burnout. Devoting to wellness strategies and support networks prior to palpable disengagement or evident exhaustion may help address rates of turnover related to burnout and may highlight the institutional dedication to the faculty.

## Limitations

Limitations of this study include a relatively small sample size from a single institution. Moreover, this study included perceptions of a sub-population of faculty (College of Health Sciences). The nature of the questions and the lack of anonymity likely resulted in fewer faculty members participating in the qualitative aspects of this investigation. The respondents provided their email addresses to receive the incentive for completing the survey; participation may have increased if an anonymous survey tool was utilized where their email was not given. Additionally, the investigators were peers of the participants, which may have impacted responses or the lack thereof. The unequal sample size between the quantitative findings and the narrative qualitative responses may not have fully captured why burnout occurred and its subsequent effect on well-being. Future studies should cast a wider net of faculty and administrators, including numerous institutions of varying sizes and missions. However, the findings of this study do contribute to the literature on higher education faculty burnout and provide possible explanations as to why this was experienced within the confines of a global pandemic.

## Conclusion

Pandemic-related factors added an additional layer of stress to nearly every profession. However, some noteworthy aspects of the responsibilities of Health Sciences faculty teaching at a mid-size, four-year institution may have contributed to a higher than anticipated degree of burnout symptoms. This analysis provides insight into why health science faculty may be experiencing feelings of disengagement and exhaustion in their work. Enhanced workload and lack of community during the pandemic were major drivers of this phenomenon. Flexibility in workload, genuine concern and appreciation expressed by institutional leaders, and accessible wellness opportunities may help to offset these negative feelings. To address faculty burnout and potential turnover, institutions of higher education must allocate resources to support faculty wellness and retention. Though logistical and monetary barriers may exist in the implementation of these strategies, an examination of the potential impact on institutional outcomes related to the well-being of its employees should be an administrative priority.
